# Shut Your Mouth

**Published:** 1870-06

**Authors:** 


Shut Your Mouth.—Professor Tyndall's researches on "Dust and Haze" continue to attract a great deal of attention, both in England and in this country. Scientific men are discussing them, and the popular press is making the most of the theme and its suggestions. The hygienic question of the hour is not what we shall eat or drink, but what —or rather how—we shall breathe. If the seeds of disease are everywhere floating in the air, and can not be removed except by filtering it through fire or water, or some such substance as cotton wool, what is to be done? Shall we all put on "respirators," and go about like so many muzzled dogs? If the only alternative is death or that sort of disfigurement, all good-looking people at least will make their wills and resignedly await their doom. But fortunately we need not bandage our mouths with filters of cotton in order to escape the poison that lurks unseen in the air. Every man who has a nose has a natural "respirator" which is all sufficient for ordinary purposes. The breath of life was breathed into man's nostrils, as we read in holy writ, and through his nostrils, and not through his mouth, it was intended that he should breathe. Some ten years ago, Catlin, of Indian notoriety, published a little book (it is still in print, we believe), entitled "*The Breath of Life*," which was a quaint but very sensible disquisition on the injurious effects of "mal respiration"—that is, mouth-respiration. It ought to be circulated as a sanitary tract, and just at this time it would be likely to be generally read. Its text, or motto, is "Shut your Mouth!" and, as the author urges, it ought to be inscribed "in every nursery and on every bedpost in the universe." It is unquestionably true that people who live and breathe according to this simple law are less liable to infectious diseases and pulmonary difficulties than those who make the mouth the main organ of respiration. Catlin gives an account of a voyage he made between two South American ports, when thirty out of eighty passengers died of yellow fever. He says that careful observation satisfied him that, with scarcely an exception, the victims of the disease were those who habitually breathed through the mouth. In numerous other instances in which he was in the midst of yellow fever and cholera, he remarked the same general exemption from disease on the part of those who kept their noses open and their mouths shut. This is worth remembering, and our readers may be sure that, even if it does not benefit them, it certainly can not harm them, to cultivate the habit of breathing through the nostrils, especially when exposed to a malarious or pestilential atmosphere. If, as Prof. Tyndall believes, ordinary air is never free from organic pollution, let us make fair trial of the nasal filters with which Nature has furnished us before we resort to any artificial contrivances for the same purpose. It will be both cheaper and more comely to shut the mouth than to muzzle it.

---

Editors Register: I am glad to see that the Missouri *Dental Journal* has an editor whose business it is to see that in each No. of the Journal we get something upon the subject of "Mechanical Dentistry," and I hope in the future to see more in the Register upon this part of Dental practice. 'Tis true that the editors of the Register have lived for some time in such an impure mechanico Dental atmosphere that they may be excusable for being disgusted with the laboratory and its productions, still they must remember that this valuable monthly visitor goes many places where to speak of a man as a mechanical Dentist is not to call him a charlatan.

In the April No. of the Register I give my experience with aluminum plate, and Starr's solder. My opinion is the same now as then, and while I would not do without Starr's solder for four times what it costs to get the right to use it, I have found that Watt and Williams, of Xenia, Ohio, have that which no Dentist can afford to do without. I refer to their alloy as an attachment for teeth to either aluminum or platinated silver plate; but I do not use it so much with aluminum as with platinated silver, as with the former. It does not unite, but with the latter a perfect union of the metals is obtained, as where gold or silver is used with their solders; but this alloy I believe to be the only one which can be used to unite block teeth to platinated silver plates where galvanic action is small or nothing. Having used, by mistake, another metal having many properties in common with that of Watt & Williams', I found I could produce the same appearing work ; but when inserted as an artificial denture turned into a galvanic battery, which set the patient's (a woman's) tongue to going at lightning speed, but all was set right when, after discovering the cause of the trouble, I used W. & W.'s metal. There being no perceptible electric action with it, also perfect cast plates can be made, and having used a number of different metals for this kind of work, I now, on account of price, purity and ease of management, use it alone. Other metals are very good, but I prefer it. I will, before closing, say that Dr. Weston, of Towanda, Pa., has constructed a flask for the purpose of making cast plates that is the best I have seen, and no one need ever make a failure using this flask with proper care. W. M. H.

ZANESVILLE.

